# Changes in the Quality and Microflora of Yellowtail *Seriola quinqueradiata* Muscles during Cold Storage

**DOI:** 10.3390/foods13071086

**Published:** 2024-04-01

**Authors:** Shota Tanimoto, Yuka Hirata, Shinta Ishizu, Run Wang, Ayumi Furuta, Ryota Mabuchi, Genya Okada

**Affiliations:** 1 Faculty of Regional Development, Prefectural University of Hiroshima, Hiroshima 734-0003, Japan; furuta@pu-hiroshima.ac.jp (A.F.); g-okada@pu-hiroshima.ac.jp (G.O.); 2 Faculty of Human Culture and Science, Prefectural University of Hiroshima, Hiroshima 734-0003, Japan; y.hrt3723@gmail.com; 3 Graduate School of Comprehensive Scientific Research, Prefectural University of Hiroshima, Shobara 734-0003, Japan; shinta0325football@docomo.ne.jp (S.I.); wrr373450054@gmail.com (R.W.); 4 Faculty of Bioresource Sciences, Prefectural University of Hiroshima, Shobara 727-0023, Japan; mabuchi@pu-hiroshima.ac.jp

**Keywords:** dark muscle, next generation sequencing, ordinary muscle, *Pseudomonas*, volatiles, storage

## Abstract

We evaluated the changes in the quality and microflora of yellowtail flesh cold-stored until spoilage. Based on the sensory evaluation, odor palatability was deemed unacceptable for dark muscle (DM) and the dorsal part of the ordinary muscle (OD) after >10 days and 14 of storage, respectively. Log 7 CFU/g in DM as well as OD was obtained on days 10 (*Aeromonas* spp.) and 14 (Enterobacteriaceae and *Pseudomonas* spp.) of storage, whereas log 5 (*Brocothrix thermosphacta*) and 6 (H_2_S-producing bacteria) CFU/g in them were obtained on day 14 of storage. In these bacteria, the viable bacterial counts of *Pseudomonas* spp. and *Aeromonas* spp. in DM were significantly higher than those in OD only at some storage times. Amplicon sequencing revealed that in both muscles, *Pseudomonas* became predominant after storage, with greater than 90% recorded after more than 10 days of storage. The relative abundances of Acinetobacter, Unclassified *Gammaproteobacter*, and *Shewanella* were relatively high in both muscles after more than 10 days of storage; however, these values were less than 5%. Ethyl butyrate in the OD and DM and 2,3-butanedione in the OD were first detected on days 14 and 10 of storage, respectively. Acetoin in the OD increased by 81-fold after 14 days of storage and was significantly increased in the DM after more than 10 days compared with the amount detected pre-storage. Volatiles, such as (*E*)-2-pentenal in the OD and 1-pentanol in the DM, decreased and increased linearly, respectively, throughout the 14-day storage period. Altogether, these volatile components may cause quality deterioration due to spoilage and/or lipid oxidation during cold storage of the OD and DM.

## 1. Introduction

Fish is widely eaten owing to rich content of protein and polyunsaturated fatty acids (PUFAs), and delicious flavor [1]. Therefore, fish consumption is encouraged to foster a healthy lifestyle. The relatively short preservation period of postmortem fish is due to the combined effects of chemical reactions, endogenous enzymes, and spoilage bacteria, which result in quality deterioration [2].

Microbial proliferation during raw seafood preservation is an important food hygiene issue [3]. Extensive research has been conducted on the growth of bacteria in fish meat during storage, but most of the studies have been measured mainly by the colony count method. Such method may underrate microbial diversity in food during storage owing to the uncultivated bacteria. It may not reveal the bacterial community that contributes to food spoilage [4,5,6,7]. Next-generation sequencing (NGS) enables precise evaluation of bacterial taxa, including uncultivated microbes or those with small proportions [8]. NGS-based analyses of raw fish spoilage have been increasing, and the results of these analyses have been published [9]. In these studies, *Psychrobacter* and *Photobacterium* were identified as the predominant genera, in addition to *Pseudomonas* and *Shewanella*, which are predominant during spoilage [9,10,11].

The volatile changes in various fish species stored before the occurrence of spoilage have been examined [12,13,14,15,16,17]. Secondary oxidation products, such as several carbonyl compounds generated by the action of lipoxygenase and the autoxidation of PUFAs, are the major volatile compounds in stored fish flesh. Of note, these carbonyl compounds may be responsible for the distinctive smell of fish [18]. In addition, trimethylamine (TMA) was the cause of the smell of fish in seawater [19], and *Shewanella* spp. is involved in its production [20]. However, carbonyls instead of TMA, are responsible for the increased fishy smell during storage before the occurrence of spoilage [13,14]. In contrast, several volatiles linked to microbial activity, except TMA, are significantly altered and have been proposed as markers of fish spoilage [10,21,22,23].

Yellowtail *Seriola quinqueradiata* is a cultured important fish species for domestic consumption and export in Japan. The ordinary muscle (white) of the yellowtail is consumed alone or in combination with the dark muscle (red, DM) in Japanese cuisine. Therefore, the changes in odor components and bacterial flora in the ordinary muscle and DM of stored yellowtail flesh should be clarified. To the best of our knowledge, these changes have not been assessed in yellowtail meat that is stored until spoilage.

Food quality has received a great deal of attention from governments, researchers, and consumers. However, rapid, reliable, and objective assessment of food quality has not yet been possible [24]. Therefore, it is necessary to investigate the quality changes in foods including fish meat discussed in this study from various perspectives in order to discover appropriate candidates for quality markers.

The purpose of the present study was to clarify the changes in the quality and bacterial flora of yellowtail meat stored in a refrigerator until spoilage using gas-liquid chromatography-mass spectrometry (GCMS) and NGS analyses. We also measured the viable bacterial count (VBC) in various media, two freshness indices (total volatile basic nitrogen (TVB-N) and TMA), and an oxidation index (thiobarbituric acid-reactive substances (TBARS)). In addition, the sensory characteristics of yellowtail flesh were evaluated.

## 2. Materials and Methods

### 2.1. Sample

Three cultured yellowtails were obtained from a local market in Hiroshima, Japan. The mean and standard deviation of weight was 3.94 ± 0.1 kg. After the fish were instantly slaughtered at the market, they were carried to the lab on ice within 8 h. Thereafter, the fish were filleted and rinsed with ice-cold 1% NaCl solution. The fillets were dissected into 1.0 cm thick sections perpendicular to the lateral line. Each section was tightly wrapped in a polyethylene film (0.01 mm thick, UBE FILM, Ltd., Tokyo, Japan) to prevent surface drying. After 0, 3, 7, 10, and 14 days of storage at 4 °C, the slices were cut to obtain DM and the dorsal part of the ordinary muscle (OD). Each divided sample was chopped with a food processor (MK-K60P; Panasonic, Osaka, Japan) and air-blast frozen in a freezer (iRiNOX AL-5M, FMI Co., Ltd., Tokyo, Japan). After the processes were repeated three times, three OD and three DM samples were prepared (*n* = 3). In other words, each of the 3 yellowtail were used to conduct the experiment at 3 different times. All prepared samples were stored at −80 °C until analysis, except for VBC.

### 2.2. Viable Bacterial Count

Each sample (5 g) was homogenized with 45 mL of sterile peptone saline (0.1% polypeptone, 0.85% NaCl) for 2 min. The samples were then diluted in series using the above saline. Briefly, 0.1 mL of the diluent was used to enumerate the heterotrophic marine bacteria, H_2_S-producing bacteria, *Pseudomonas* spp., and *Aeromonas* spp. The diluent was then spread onto the respective culture media. Heterotrophic marine bacteria were incubated on marine agar (Condalab, Madrid, Spain) at 20 °C for 72 h. Black colonies of H_2_S-producing bacteria on iron agar (Condalab) with 5.0% NaCl were enumerated after 96 h of incubation at 25 °C. *Pseudomonas* spp. were incubated on cephaloridine fucidin cetrimide agar (CFC) (Merck KGaA, Darmstadt, Germany) with 1.0% (*v*/*v*) glycerol at 35 °C for 72 h. *Aeromonas* spp. were incubated on Aeromonas isolation agar (Sigma-Aldrich, St. Louis, MO, USA) with ampicillin (5.0 mg/L) at 20 °C for 72 h. Thereafter, 1.0 mL of diluent was used to enumerate the total bacterial counts (total bacteria), lactic acid bacteria, *Brochothrix thermosphacta*, and Enterobacteriaceae. Plate count agar (0.50% peptone, 0.50% NaCl, 0.25% yeast extract, 0.10% glucose, 1.50% agar, pH 7.0 ± 0.2) for total bacteria was incubated at 30 °C for 72 h. De Man, Rogosa, Sharpe agar (Merck KGaA) for lactic acid bacteria was incubated at 25 °C for 72 h. Streptomycin-thallous acetate-actidione agar for *Brochothrix thermosphacta* was incubated at 25 °C for 48 h [25]. Violet Red Bile Glucose agar (Merck KGaA) for Enterobacteriaceae was incubated at 25 °C for 48 h. The diluent was then mixed with the corresponding culture medium and cultured. The colony-forming unit per g of sample (cfu/g) was used for the VBC.

### 2.3. Biochemical Measurements

For TVB-N and TMA, 1.0 g of each sample was homogenized with 10 mL of 5% trichloroacetic acid (TCA). After centrifugation for 20 min at 10,000× *g*, TVB-N was determined via microdiffusion analysis, according to Conway [26]. Briefly, 1.0 mL of the TCA extract was mixed with saturated Na_2_CO_3_ and incubated at 37 °C for more than 80 min. The TVB-N was then trapped in a 1% boric acid solution. This solution was titrated with 10.0 mM H_2_SO_4_. Finally, the TVB-N (mg N/100 g) was determined by the titration volume of the sample and blank (1 mL of 5% TCA).

TMA was determined using a GCMS system according to the method of Hamakawa et al. [27]. In brief, 2.0 mL of the TCA extract, 100 µL of 2.0 mg/mL triethylamine (internal standard solution), and 1.0 mL of 65% KOH were mixed and shaken for 30 s. After the mixture was heated at 30 °C for 10 min, 1.0 µL of the upper layer was employed for a GCMS (GCMS-QP2010, Shimadzu, Tokyo, Japan). GC was achieved using a column (Rtx-Volatile amine, 30 mm × 0.32 mm, RESTEK, PA, USA) and the following parameters: carrier gas, helium; column flow rate, 50 cm/s; and oven temperature, initially 35 °C for 10 min, increased at 20 °C/min to 250 °C, and held at 250 °C for 5 min. The mass spectrometry was performed in electron impact mode at 70 eV. The ions monitored in the selected ion monitoring mode were *m*/*z* 42, 58, and 59 for TMA and *m*/*z* 58 and 86 for internal standard. The injection port, ion source, and transfer line were set at 250 °C, 200 °C, and 250 °C, respectively.

TBARS were measured according to the method of Mukojima et al. [28].

### 2.4. Solid Phase Micro Extraction and Gas Liquid Chromatography-Mass Spectrometry

Volatiles were extracted by SPME (solid phase micro extraction) according to our previous study [25] but as follows. A frozen sample (1.2 g), 6.0 mL of saturated NaCl solution, and an SPME fiber coated with 65 μm polydimethylsiloxane/divinylbenzene (Supelco Co., Bellefonte, PA, USA) were used for volatile extraction. The supernatant (5.0 mL) after centrifugation at 10,000× *g* for 10 min at 4 °C was incubated for SPME.

The extracted volatiles were measured using GC according to our previous study [28] but as follows. GCMS transfer lines were maintained at 200 °C. Masses range and scan rate were set at 40–200 *m*/*z* and 2.0 scan/s, respectively. The splitless injection for 1.0 min was performed under cryofocusing, followed by split mode for 5.0 min.

### 2.5. Sensory Test

A sensory test was performed by 42 trained panels. The participants were graduate and undergraduate students of the Prefectural University of Hiroshima, Japan. A 5 g frozen sample in a vial (20 mL) was allowed to thaw at room temperature (approximately 25 °C). The samples were evaluated after 2 h. The panelists judged the intensity of the putrid odor and odor palatability of the fish muscles using a 5-point scale: putrid odor 0, no smell; 1, very weak; 2, weak; 3, normal; 4, strong; 5, very strong; and odor palatability −2, very unfavorable; −1, unfavorable; 0, normal; 1, favorable; 2, very favorable. For the palatability, a sample considered unacceptable by the panelists was scored as −1 or lower. Prior to this evaluation, the subjects were given full informed consent to protect their human rights. In addition, we have complied with the Ethical Guidelines for Medical and Biological Research Involving Human Subjects, the Act on the Protection of Personal Information, and other applicable laws, regulations, and ordinances. Furthermore, we took the utmost precautions to prevent food poisoning [29,30].

### 2.6. DNA Extraction and Next-Generation Sequencing

DNA extraction was conducted according to our previous study [31]. but as follows. The bacterial pellet was resuspended in 500 μL of physiological saline (0.85% NaCl). Quick-DNA™ Fecal/Soil Microbe Miniprep Kit (Zymo Research Corporation, Tustin, CA, USA) was used to extract microbial DNA from the suspension.

PCR and NGS of the V3–V4 region of the bacterial 16S rRNA gene were performed according to our previous study [31], except that the first PCR was carried out for 30–40 cycles.

### 2.7. Sequencing Data

The raw fastq data have been deposited with links to PRJNA1065935 in the NCBI. Metagenomic analysis of raw short reads was performed using QIIME 2 software (2019.7) and the statistical software package, R (Ver.4.0.4) according to our previous study [31] except as follows. A hierarchical cluster analysis (HCA) heatmap of the relative abundance of microbial genera was generated using Metaboanalyst 5.0 and the Ward method [32]. Data were corrected for the second-lowest relative abundance and converted to common logarithms before processing. The 51 genera with a relative abundance greater than 0.01 in any of the samples were selected for the analysis.

### 2.8. Statistical Analyses

Duncan’s new multiple-range test and Student’s *t*-test or Welch’s test were applied using SPSS Statistics (version 17.0; IBM Japan, Ltd., Toyko, Japan) to determine the differences between the mean values of the samples. Before statistical analysis, the data for VBC were converted to log10 CFU/g. SIMCA 16 software (Sartorius AG, Göttingen, Germany) was used for principal component analysis (PCA) of the volatiles.

## 3. Results and Discussion

### 3.1. Viable Bacterial Count

The changes in the VBCs of yellowtail muscle in various culture media during storage are shown in Table 1 and Table S1. Total bacteria and marine bacteria in both the OD and DM stored for more than 6 days increased significantly compared to those detected before storage. In the OD and DM, these bacteria exceeded 7 Log CFU/g, which was the starting point of fish meat decomposition [33], on days 10 and 14 of storage, respectively. *Aeromonas* spp., *Pseudomonas* spp., and Enterobacteriaceae in both the OD and DM increased significantly after 3, 6, or more days of storage compared to those detected before storage, reaching seven orders of magnitude on day 10 or 14 of storage. The levels of *Brochothrix thermosphacta*, a spoilage-causing bacterium in meat, and H_2_S-producing bacteria were less than one order of magnitude before storage in both muscle types (*p* < 0.05). These bacteria propagated significantly during storage but increased by five or six orders of magnitude on day 14 of storage. A significant change in lactic acid bacteria was observed in the DM sample; however, this increase was only one order of magnitude. The results derived using the culture-dependent method suggest that regardless of the muscle type, yellowtail meat spoils on day 10 under refrigerated conditions, with *Aeromonas* spp., *Pseudomonas* spp., and Enterobacteriaceae as the major spoilage bacteria. In general, the spoilage microflora in stored fish under aerobic conditions are *Aeromonas*, *Pseudomonas*, and *Shewanella* [3], supporting the predominant bacteria of post-spoilage yellowtail muscles in the present study. Enterobacteriaceae were also found in the spoilage microflora of some fish during refrigerated storage; however, their VBCs were always lower than those of the pseudomonads [34,35].

### 3.2. Biochemical Measurements

The changes in the TVB-N, TMA, and TBARS values of the yellowtail muscle during storage were shown in Table 2 and Table S2. TVB-N in the OD and DM stored for more than 10 and 6 days increased significantly compared to that prior to storage, respectively. The OD had a significantly higher TVB-N than the DM during storage, except on day 10. TMA in the OD and DM increased significantly after more than 10 and 6 days of storage, respectively, in comparison to that prior to storage. The TMA in the DM indicated higher levels compared to those in the OD during storage (*p* < 0.05). The TVB-N levels in the OD and DM did not exceed 25–35 mg/100 g, an indicator of spoilage [36,37], after storage. The acceptable limit of TMA for various fish species is 42–63 µg/g [36,38]. However, the TMA values in the OD in the present study did not surpass this limit during storage. After more than 6 days of storage, the TMA level in the DM exceeded the limit. Therefore, TVB-N in the OD and DM, and TMA in the OD are considered insufficient indicators of yellowtail meat spoilage under the present refrigerated conditions, whereas TMA in the DM is a sufficient indicator of such spoilage. TMA is produced from trimethylamine oxide (TMAO) by bacteria, such as Aeromonas spp., *Pseudomonas phosphoreum*, psychrotolerant Enterobacteriaceae, *Shewanella putrifaciens*, and *Vibrio* spp. during seafood spoilage [39,40]. Based on the VBC in addition to NGS results described below, *Shewanella* and *Pseudomonas* may be involved in the increase in TMA during the latter stage of storage. Yellowtail DM is reported to contain approximately twice as much TMAO as the OD [41]. The DM of tuna, bonito, and sardine is presumed to include TMAO reductase [42,43]. Therefore, the yellowtail DM may exhibit TMAO-reducing activity.

The TBARS in the OD and DM stored for more than 10 and 6 days showed significantly higher values compared to those prior to storage, respectively. During storage, the DM had significantly higher TBARS levels compared to the OD. These align with the changing behavior of TBARS in yellowtail meat stored at 5 °C prior to spoilage [44]. However, no significant increment in TBARS was found during the latter part of storage when the fish meat was spoiled. Moreover, prolonged storage after spoilage may have resulted in further degradation of the secondary lipid oxidation products. Viji et al. reported a decrease in TBARS in catfish during storage, which was due to the interaction of TBARS with muscle components [45]. Therefore, TBARS may not be a suitable indicator of the wide range of quality changes in yellowtail meat (i.e., from deterioration in freshness to spoilage).

### 3.3. Volatile Compounds by Solid Phase Micro Extraction and Gas Liquid Chromatography-Mass Spectrometry

The changes in the volatile compounds in yellowtail muscle during storage are shown in Table S3. During storage, 155 peaks were detected and 138 compounds (40 aldehydes, 28 alcohols, 24 ketones, 24 hydrocarbons, nine fatty acids, six furans, two esters, two S-containing compounds, one N-containing compound, and one lactone) were identified. The number of volatile compounds, which decreased in the OD in comparison to that prior to storage (*p* < 0.05), increased from 21 on day 3 to 47 on day 14 of storage, and that of increased volatile compounds (*p* < 0.05) varied from 3 on day 3 of storage to 35 on day 14. The number of volatiles that showed a significant decrease in the DM in comparison to the pre-storage level varied from 1–3 during storage, whereas that of increased volatile compounds (*p* < 0.05) increased from 4 on day 6 of storage to 82 on day 10 and decreased to 46 on day 14. The number of volatiles, which was significantly higher in the DM than the OD, increased from 9 prior to storage to 60 on day 14. The number of volatiles, which was significantly lower in the DM than in OD, decreased from 47 before storage to 1–10 after storage. These indicate that the changing pattern of volatiles in yellowtail flesh varies with the type of muscle. These results resembled those of cold-stored yellowtail flesh without spoilage in our previous study [44]. These differences are complicated by various factors, such as differences in the activity of enzymes involved in lipid oxidation, antioxidant systems, myoglobin content between the OD and DM, and bacterial metabolism associated with spoilage [46,47,48,49]. However, the mechanism underlying volatile changes in fish meat during storage requires further investigation.

Ethyl butyrate was first detected on day 14 of storage in both OD and DM, suggesting that spoilage microorganisms contributed to the formation of this compound. Acetoin and a compound with a KI of 1318 in the OD increased by 81- and 228-fold after 14 days of storage from less than 0.1 ng/g before storage and were significantly increased in the DM stored for more than 10 days relative to the pre-storage levels. Therefore, these compounds are useful markers of yellowtail meat spoilage. Most volatile compounds that rose significantly after storage in the OD were hydrocarbons, such as terpenes, alkanes, and aromatics. However, the reason for the increased levels of these components during storage is unclear. 2,3-Butanedione in the OD remained undetectable until day 6 of storage and increased significantly after more than 10 days of storage. Of the compounds that changed significantly during storage, some volatiles, such as (*E*)-2-decenal and (*E*)-2-pentenal in OD, and 1-pentanol and 1-hexanol in the DM, decreased and increased linearly throughout the 14-day storage period, respectively, and are potential quality degradation indices for the OD and DM during cold storage. *Pseudomonas* in meat is involved in the production of various volatiles, such as aldehydes, alcohols, esters, ketones, and S-containing compounds [34,50]. Volatiles, such as benzaldehyde, 2,4-di-tert-butylphenol, and 1-hexanol, have been proposed as spoilage markers caused by *Acinetobacter johnsonii* XY27 in bigeye tuna [51]. 2-heptanone, 2-nonanone, 1-Octen-3-ol, methanethiol, dimethyl disulfide, and TMA are the key volatile compounds in tuna inoculated with *Shewanella putrefaciens* [21,52]. These genera, which were detected using culture-dependent methods or NGS, may contribute to the formation of volatile components related to spoilage as described below. In the future, these bacteria should be isolated and the changes in volatile components after their inoculation into fish flesh should be analyzed to confirm whether the production of these candidate markers is due to bacterial action.

**Figure 1:**
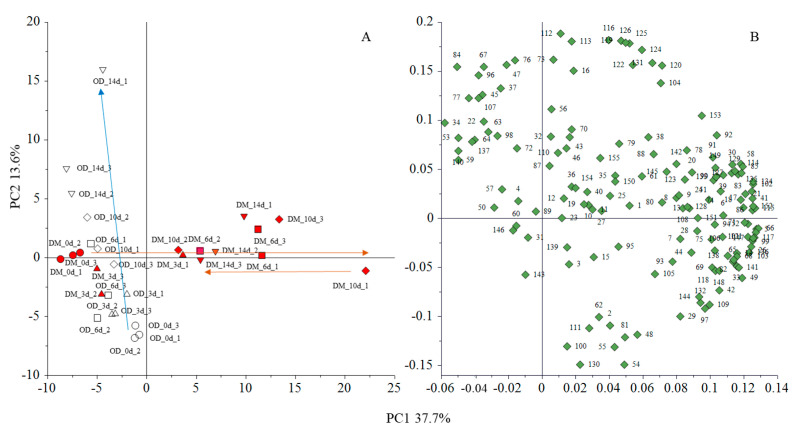
Score scatter plot (**A**) and loading plot (**B**) of principal component analysis (PC1 and PC2) based on the volatiles in raw yellowtail muscles during storage. The data were pre-processed by auto-scale. A; OD, the dorsal part of the ordinary muscle. DM, dark muscle. Storage time is indicated as ex. 14 d (storage for 14 days). The numbers after the storage time represent the sample number. B; The numbers in the loading plot indicate peak number in Table S3. Arrows in the score scatter plot indicate changes in OD (blue) and DM (red) during storage.

The PCA results of the volatiles of yellowtail flesh during storage are shown in Figure 1 and Tables S4 and S5. The scores for PC1 and PC2 comprised 37.7% and 13.6% of the total variation, respectively. First, PC1 distinguished the stored DM samples, except for some of the day 3 samples, from those of all OD and pre-storage DM samples. For DM, although some variations were found among the samples, the PC1 score increased as storage proceeded for up to 10 days; however, the score on day 14 of storage decreased. In contrast, the ODs stored for long periods had higher PC2 scores. Therefore, the PCA score results show that the change patterns of volatile component compositions are remarkably different between DM and OD. In addition, these results demonstrated that in DM, the volatile components that increased during storage decreased with further storage. Of the many volatile compounds that had positive loadings for PC1, aldehydes, such as 1-octen-3-ol and (*E*)-2-hexenal, and unknown compounds, such as that with a KI of 2011, had higher factor loadings (Table S3). These volatiles were responsible for variations in DM during storage and the gap between OD and DM with increasing storage period with some exceptions. For PC2, unknown compounds, such as that with a KI of 1538, and hydrocarbons, such as styrene, had higher positive factor loadings; however, aldehydes, such as (*E*)-2-decenal, had lower negative factor loadings. Thus, for the OD, these changes were responsible for the PC2 scores during storage. Thus, the volatile components as noted above contribute to the differences in the changing patterns of volatile components between OD and DM during storage.

### 3.4. Sensory Test

**Figure 2:**
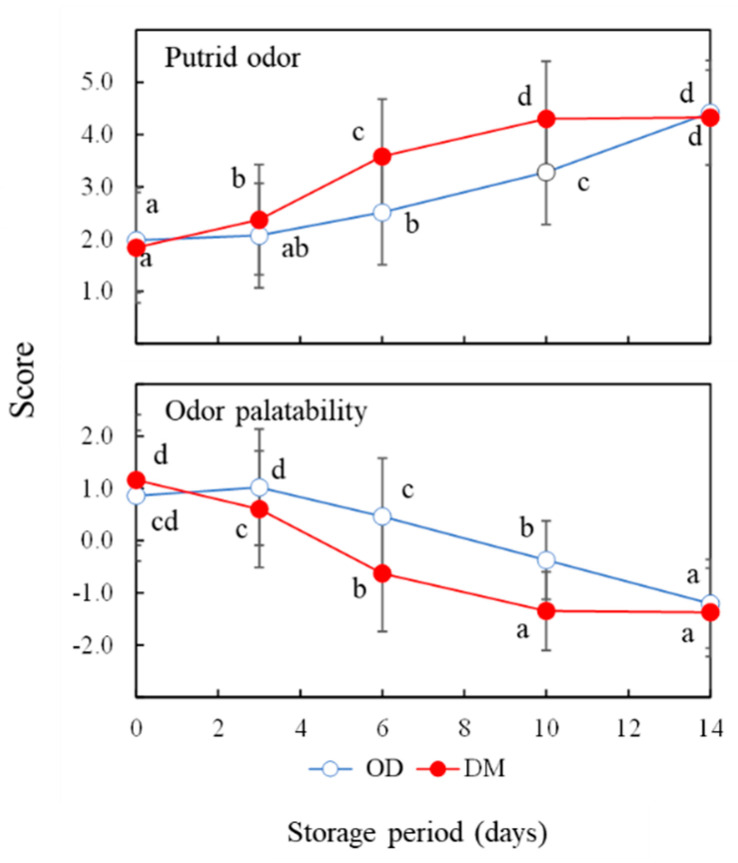
Sensory test of raw yellowtail muscles during storage at 4 °C. Values and error bars indicate the means and standard deviations of scores on a 5-point scale (1 to 5 for putrid odor and −2 to 2 for odor palatability). OD, the dorsal part of the ordinary muscle; DM, Dark muscle. Different lowercase letters for the same muscle type and odor characteristics indicate significant differences.

The sensory test of the odor of yellowtail muscles was performed (Figure 2). The scores of putrid odor and odor palatability of the OD increased and decreased significantly after more than 6 and 10 days of storage, respectively, in comparison to those prior to storage. The scores of putrid odor and odor palatability of the DM increased and then decreased significantly after more than 3 days of storage in comparison to those prior to storage. For odor palatability, the panelist judged the sample to be unacceptable, assigning a score of −1 or less, at 14 days and 10 days or more of storage for the OD and DM, respectively. These mean that the odor of the DM deteriorated faster during cold storage than that of the OD.

### 3.5. Next-Generation Sequencing

**Figure 3:**
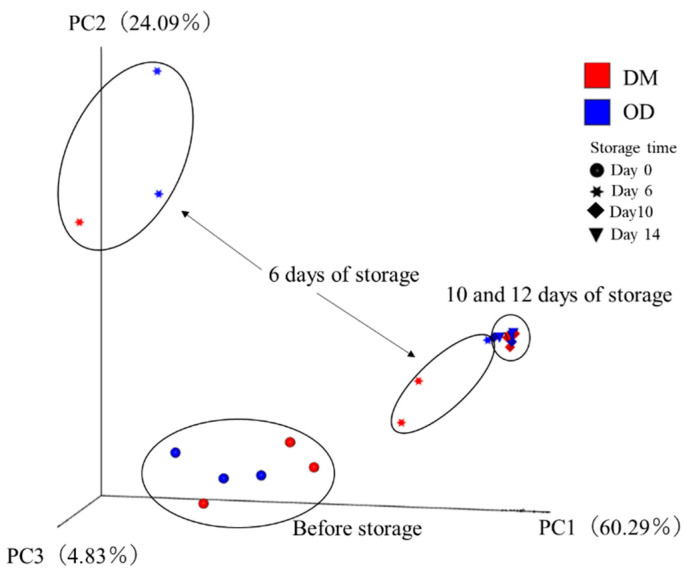
Principal coordinate analysis based on microbial community in yellowtail muscles at the genus level during storage at 4 °C. OD, the dorsal part of the ordinary muscle; DM, Dark muscle. Analysis was performed using the weighted UniFrac method.

Table 3 and Table S6 show the changes in the alpha diversity index of yellowtail flesh during storage. Good’s coverage index was greater than 0.99 for all samples, indicating that the sequence data provided adequate coverage of the microbial communities under investigation. The operational taxonomic unit number, Chao 1 index, and ACE index of the DM before storage had significantly higher values in comparison to those after 10 days or more of storage. The Shannon and Simpson indices of the OD and DM stored for more than 6 days or more had significantly lower values in comparison to those preserved for 0 and 6 days. Combined with the VBC results (Table 1), the diversity of the bacterial flora can be inferred to be lost with fish meat spoilage, regardless of the muscle type. The PCoA results of the bacterial communities in yellowtail flesh during storage are shown in Figure 3. The PCoA scores of the first three principal components (PCo1, PCo2, and PCo3) accounted for 60.29%, 24.09%, and 4.83% of the total variation, respectively. The PCoA scores of the OD and DM were assigned to three groups: 1: Samples before storage; 2: Samples stored for more than 10 days, including a batch of samples from day 6 of storage; and 3: Other samples. Samples stored for more than six days and samples before storage were located close to each other, especially the former. On the other hand, the PCoA score did not show a good separation between muscles of the same storage time. The PCoA suggested that the microbiota of yellowtail flesh was different prior to storage and after the long-term storage, regardless of muscle type.

**Figure 4:**
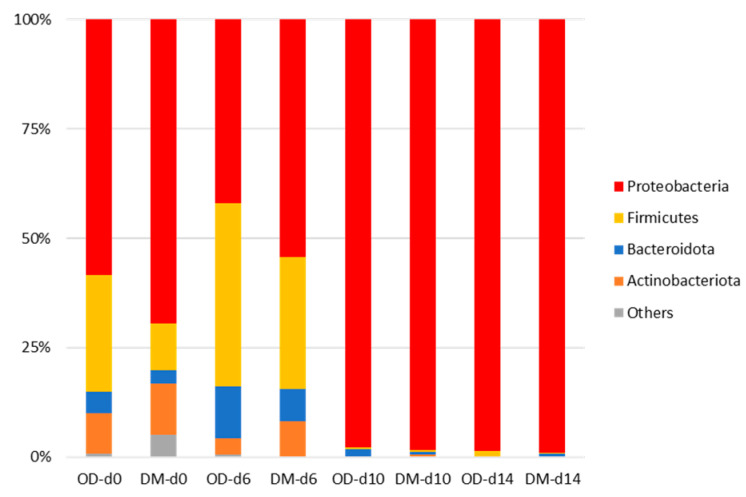
Relative abundance at the phylum level based on the classification of partial 16S rDNA sequences of bacteria from yellowtail muscles during storage at various temperature for 12 days. OD, the dorsal part of the ordinary muscle; DM, Dark muscle. The storage time is indicated by d#.

**Figure 5:**
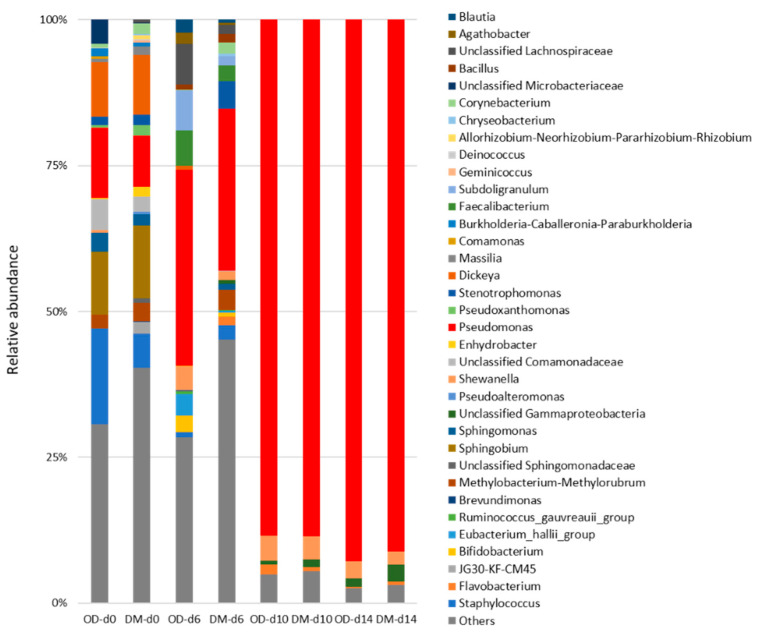
Relative abundance at the genus level based on the classification of partial 16S rDNA sequences of bacteria from yellowtail muscles during storage at 4 °C for 14 days. OD, the dorsal part of the ordinary muscle; DM, Dark muscle. The storage time is indicated by d#. OD, the dorsal part of the ordinary muscle; DM, Dark muscle. The storage time is indicated by d#.

Figure 4 and Figure 5 summarize the taxonomic classification of the yellowtail muscle microbial community during storage at the phylum and genus levels, respectively. Proteobacteria was the most dominant phylum in the OD and DM before storage, followed by Firmicutes and Actinobacteria. In both muscles, the relative abundance of Proteobacteria exceeded 95% after more than 10 days of storage. In contrast, no predominant genera were observed in either muscle before storage, and only *Dickeya*, *Pseudomonas*, *Staphylococcus*, and *Sphingobium* showed relative abundances of more than 5%. Among these genera, only *Pseudomonas* was found in the bacterial flora of fish caught in clean, unpolluted waters [53]. *Pseudomonas* became predominant after storage, with the percentage of this genus reaching more than 90% after more than 10 days of storage. The relative abundances of *Acinetobacter*, Unclassified *Gammaproteobacter*, and *Shewanella* were relatively high in both muscles stored for more than 10 days; however, these values were less than 5%. *Pseudomonas* spp. exceeded 7 Log CFU/g in both OD and DM after more than 10 days of storage (Table 1), which was consistent with the NGS results. Dimitrios et al. and Parlapani have reported that *Pseudomonas* was a spoilage-causing microorganism in many fish species based on culture-independent and culture-dependent methods [9,11]. Dimitrios et al. have reported that *Pseudomonas* and *Shewanella* induced the spoilage of fish from a temperate sea area owing to these phenotypic approaches [9]. Five and 7 Log CFU/g of H_2_S-producing bacteria (mainly *Shewanella putrefaciens*) were found in both the OD and DM on days 10 and 14, respectively (Table 1). These results aligned with those of the present study, in which the relative abundance of *Shewanella* at the latter part of storage indicated lower values in comparison to that of *Pseudomonas*. *Acinetobacter* spp. is dominant in the flesh of some cold-stored fish [2]. *Aeromonas* spp. may have exceeded 7 Log CFU/g in both the OD and DM after more than 10 days of storage (Table 1) as *Aeromonas* is a genus classified as *Gammaproteobacter* [54]. Yang et al. revealed that *Aeromonas sobria* is a spoiler of Pacific white shrimp using culture-dependent methods, but not NGS [55]. *Brochothrix* had a relative abundance of less than 1% in both muscles after more than 10 days of storage, supporting the *Brochothrix thermosphacta* counts (Table 1). In contrast, Enterobacteriaceae increased significantly in both the OD and DM during storage, reaching 7 Log CFU/g on day 14 (Table 1). However, as the NGS results revealed that the genera classified as Enterobacteriaceae were undetectable in both muscle types after storage, Enterobacteriaceae detected using culture-dependent methods may belong to other families. *Psychrobacter* can induce the spoilage of several raw fish [9,11]; however, this genus was hardly detected in chilled yellowtail muscles at the end of the storage period. In the present study, no differences in microflora changes were found between the yellowtail muscles during storage, using both culture and non-culture methods. Li et al. revealed that the dominant bacteria in cold-stored common carp meat are *Aeromonas* and *Pseudomonas*; however, the microbial flora differs slightly between the ordinary muscle and DM [21]. Therefore, in yellowtail meat, in addition to *Pseudomonas*, genera, such as *Acinetobacter* and *Shewanella*, are mainly involved in the progression of spoilage, regardless of muscle type. Further studies are needed to identify these species and gain a better understanding of yellowtail meat spoilage.

**Figure 6:**
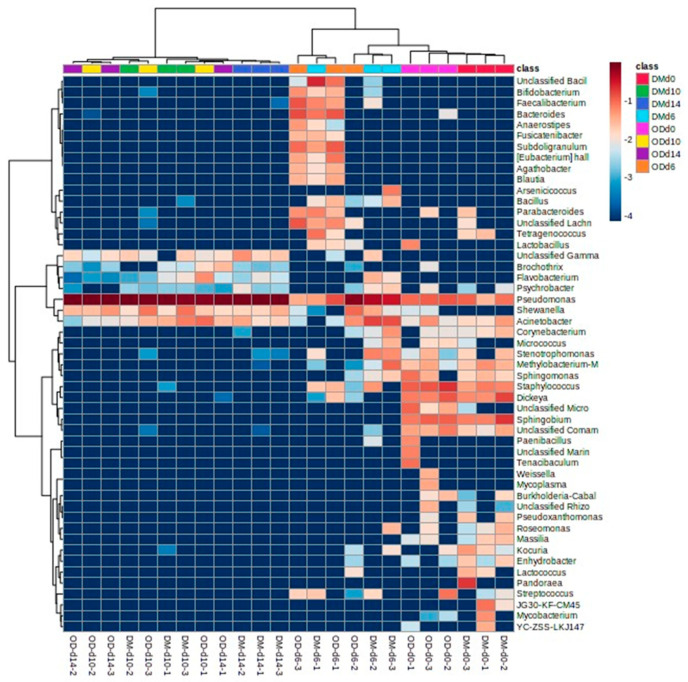
Heat map visualization and hierarchical clustering of the dataset of relative abundance at the genus level based on the classification of partial 16S rDNA sequences of bacteria from yellowtail muscles during storage at 4 °C for 14 days. OD, the dorsal part of the ordinary muscle; DM, Dark muscle. The storage time is indicated by d#. The numbers after the storage time indicate the number assigned to the sample. The class indicates the muscle type and storage time. Colors indicate the common logarithm of relative abundance.

Figure 6 shows the HCA heat map of the relative abundance of bacteria at the genus level in yellowtail muscles during storage by NGS. The HCA separated the yellowtail flesh into two major clusters. Muscles stored for more than 10 days were assigned to cluster I. The other samples were subdivided into two further clusters: II (part of the day 6 samples) and III (samples before storage and part of the day 6 samples). Three clusters were identified in the genera: cluster A included *Bacillus* and *Bifidobacterium*; cluster B included *Pseudomonas*, *Acinetobacter*, and *Shewanella*; and cluster C included *Dickeya*, *Staphylococcus*, and *Sphingobium*. The cluster for the samples and genera and the heatmap of the relative abundance of genera confirm the PCoA scores and classification of the microbiota at the genus level (Figure 3 and Figure 5).

## 4. Conclusions

Yellowtail meat was refrigerated to assess the changes in its quality and bacterial flora. After more than 10 or 14 days of storage, the OD and DM were organoleptically spoiled, respectively. After spoilage, *Pseudomonas* was predominant in both muscles, followed by *Acinetobacter*, unclassified *Gammaproteobacter*, and *Shewanella*, with a relative abundance of less than 5%. Acetoin, 2,3-butanedione, (*E*)-2-decenal, ethyl butyrate, 1-hexanol, 1-pentanol, (*E*)-2-pentenal, and a compound with a Kovats retention index of 1318 (unknown compound) in the OD and/or DM exhibited characteristic changes during storage and may have been produced by the aforementioned bacterial species. Therefore, these volatile components are potential quality deterioration markers for yellowtail meat, ultimately leading to spoilage. In the future, the bacteria responsible for producing these volatile components should be identified and the impacts of storage temperature on changing the microbiota and volatile components should be determined.

## Figures and Tables

**Table 1:** Visible bacterial counts (VBCs) of yellowtail fish muscles during storage at 4 °C (log CFU/g).

	Muscle Type	Storage Periods (Days)									
		0		3		6		10		14	
Total viable count	OD	3.0	^a^	2.5	^a^	4.0	^b^	7.3	^c†^	8.6	^d^
	DM	3.0	^a^	2.7	^a^	4.0	^b^	6.6	^c^	8.2	^d^
Heterotrophic marine bacteria	OD	1.6	^a^	2.1	^a^	4.2	^b^	7.0	^c^	8.5	^d†^
	DM	2.3	^a^	2.9	^ab^	3.4	^b^	6.6	^c^	7.9	^d^
Lactic Acid Bacteria	OD	0.6	^a^	0.6	^a^	0.8	^a^	1.2	^a^	1.2	^a^
	DM	0.3	^a^	0.7	^ab^	1.0	^ab^	1.5	^b^	1.1	^ab^
*Enterobacteriaceae*	OD	1.3	^a^	0.9	^a^	3.1	^b^	6.4	^c^	7.5	^c^
	DM	1.7	^b^	0.2	^a^	2.7	^c^	5.9	^d^	7.3	^e^
*Pseudomonas* spp.	OD	0.7	^a^	2.2	^ab^	3.5	^b†^	6.1	^c^	7.6	^c^
	DM	1.8	^a^	1.5	^ab^	2.8	^b^	5.9	^c^	7.2	^d^
*Aeromonas* spp.	OD	1.5	^a^	2.7	^b^	4.2	^c†^	7.2	^d^	8.4	^e†^
	DM	1.6	^a^	2.3	^a^	3.7	^b^	6.7	^c^	8.2	^d^
*Brochothrix* *thermosphacta*	OD	0.3	^a^	0.0	^a^	0.9	^a^	3.6	^b^	5.4	^b†^
	DM	0.0	^a^	0.0	^a^	0.5	^a^	3.7	^b^	4.8	^b^
H_2_S-producing bacteria	OD	0.0	^a^	1.2	^b^	3.1	^c^	5.1	^d^	6.5	^e^
	DM	0.0	^a^	0.6	^a^	2.9	^b^	4.8	^c^	6.1	^d^

The values indicated means ± standard deviation of triplicate determinations (*n* = 3). OD, the dorsal part of the ordinary muscle; DM, dark muscle. Daggers for the same storage days and the same mediums with different letters indicate significant differences. Small letters for the same flesh types and the same mediums with different letters indicate significant differences.

**Table 2:** Total volatile basic nitrogen (TVB-N), trimethyl amine (TMA), and thiobarbituric acid-reactive substances (TBARS) of yellowtail fish muscles during storage at 4 °C.

	Muscle Type	Storage Periods (Days)									
		0		3		6		10		14	
TVB-N (mg/100 g)	OD	14.9	^a†^	14.6	^a†^	15.8	^a†^	15.9	^B^	18.3	^b†^
	DM	10.4	^a^	11.9	^ab^	13.0	^b^	15.9	^b^	16.9	^b^
TMA (μg/g)	OD	0.21	^c†^	1.28	^c†^	1.36	^c†^	3.67	^b†^	12.23	^a†^
	DM	13.95	^d^	39.70	^cd^	69.05	^bc^	108.68	^ab^	145.31	^a^
TBARS (μmol/g)	OD	0.004	^a†^	0.008	^a†^	0.012	^a^	0.019	^b†^	0.016	^b†^
	DM	0.326	^a^	0.384	^ab^	0.935	^bc^	1.823	^cd†^	1.943	^d^

The values indicated means ± standard deviation of triplicate determinations (*n* = 3). OD, the dorsal part of the ordinary muscle; DM, dark muscle. Daggers for the same storage days and the same analyzed items with different letters indicate significant differences. Small letters for the same flesh types and the same analyzed items with different letters indicate significant differences.

**Table 3:** Alpha diversity of yellowtail fish muscles during storage at 4 °C.

	**Muscle** **Type**	**Storage Periods (Days)**							
		**0**		**6**		**10**		**14**	
OUTs	OD	51.0		49.7		22.7		23.0	†
	DM	77.0	a	56.7	ab	23.7	B	27.0	b
Chao1	OD	51.0		49.7		22.7		24.0	†
	DM	77.3	a	57.0	ab	23.7	C	27.3	bc
ACE	OD	51.0		49.7		22.7		24.0	†
	DM	77.3	a	57.0	ab	23.7	C	27.3	bc
Goods_ coverage (%)	OD	1.000		1.000		1.000		1.000	
	DM	1.000		1.000		1.000		1.000	
Shannon	OD	4.73	a	4.67	a	2.67	B	2.71	b
	DM	5.24	a	4.59	a	2.67	B	2.83	b
Simpson	OD	0.947	a	0.936	a	0.748	B	0.725	b
	DM	0.944	a	0.921	a	0.764	B	0.740	b

The values represent mean ± standard deviation of triplicate determinations (*n* = 3). OD, the dorsal part of the ordinary muscle; DM, dark muscle. Daggers for the same storage days and the same alpha diversity index with different letters indicate significant differences. Small letters for the same flesh types and the same alpha diversity index with different letters indicate significant differences.

## Data Availability

The original contributions presented in the study are included in the article/Supplementary Material, further inquiries can be directed to the corresponding author.

## Supplementary Materials

The following are available online at https://www.mdpi.com/article/10.3390/foods13071086/s1, Table S1: Visible bacterial counts of yellowtail fish muscles during storage at 4 °C (log CFU/g), Table S2: Total volatile basic nitrogen (TVB-N), trimethyl amine (TMA), and thiobarbituric acid-reactive substances (TBARS) of yellowtail fish muscles during storage at 4 °C, Table S3: Changes in volatile compounds in raw yellowtail muscles during storage at 4 °C (ng/g), Table S4: Scores of principal components analysis (PC1 and PC2) based on volatiles in yellowtail fish muscles, Table S5: Factor loadings of principal components analysis (PC1 and PC2) based on volatiles in yellowtail fish muscles during storage, Table S6: Alpha diversity of yellowtail fish muscles during storage at 4 °C.
